# Clinical Value of Kidney Immunodeposits and Urinary Complement Activation Fragments in IgAN

**DOI:** 10.1016/j.ekir.2026.106648

**Published:** 2026-06-08

**Authors:** Virginie Royal, Yasar Caliskan, Louis-Philippe Laurin, Arnaud Bonnefoy, Clémence Merlen, Krista L. Lentine, Stéphan Troyanov, CureGN Collaborators, Andrew Bomback, Ali Gharavi, Krzysztof Kiryluk, Larry Greenbaum, John Mahan, William Smoyer, Myda Khalid, Jon Klein, Michelle Rheault, Vimal Derebail, Ronald Falk, Krzysztof Mucha, Tarak Srivastava, Elif Erkan, Jill Krissberg, Mahmoud Kallash, Chloe Douglas, Elizabeth Onugha, John Barcia, Brian Stotter, Louis-Philippe Laurin, Milos Budisavljevic, Isabelle Ayoub, Jason Kidd, Ibrahim Batal, Vivette Denise D'Agati, Gabriele Gaggero, Satoru Kudose, Glen Markowitz, Dominick Santoriello, Miroslav Sekulic, Michael Barry Stokes, Valerio Vellone, Alexander Katz, Hong (Julie) Yin, Laura Biederman, Joseph Gaut, Virginie Royal, Mathieu Latour, Natlie (Natacha) Patey, Vishwajeeth Pasham, Anjali Satoskar, Anthony Chang, Huma Fatima, J. Charles Jennette, Vanessa Moreno, Agnes Fogo, Selvaraj Muthusamy, Victoria Kolupaeva, Samantha Martinek-Bundt, Dawson Carmean, Mary Dreher, Samantha Sharpe, Caroline Poulton

**Affiliations:** 1 Division of Pathology, Hôpital Maisonneuve-Rosemont, University of Montreal, Montreal, Canada; 2 Division of Nephrology and Hypertension, Vanderbilt University Medical Center, Nashville, Tennessee, USA; 3 Division of Nephrology, Hôpital Maisonneuve-Rosemont, University of Montreal, Montreal, Canada; 4 Division of Hematology, Centre Hospitalier Universitaire Sainte-Justine, University of Montreal, Montral, Canada; 5 Division of Nephrology, SSM Health Saint Louis University Hospital, Saint Louis, Missouri, USA; 6 Division of Nephrology, Hôpital du Sacré-Cœur-de-Montréal, University of Montreal, Montreal, Canada

**Keywords:** electron microscopy, immunofluorescence, light microscopy, outcomes, proteinuria, urinary sC5b9

## Abstract

**Introduction:**

Ig deposits and complement activation are central in the pathogenesis of IgA nephropathy (IgAN). Whether immunodeposits, assessed by immunofluorescence (IF) and electron microscopy (EM), or the measurement of urinary complement activation fragments, can help determine the likelihood of progression of IgAN is unknown.

**Methods:**

In this retrospective analysis of 247 patients of IgAN with pathology assessments and clinical follow-up from the Cure Glomerulonephropathy (CureGN) cohort, we assessed immunodeposits by IF (intensity and localization of IgA, C3, IgG, and IgM) and EM (quantity and localization of deposits and foot process effacement [FPE]), as well as urinary membrane attack complex at enrollment (soluble C5b9 [sC5b9]). We tested associations between immunodeposits and urinary sC5b9 with light microscopy (LM) with proteinuria and survival from kidney failure or a ≥ 40% decline in estimated glomerular filtration rate (eGFR; combined outcome).

**Results:**

Mesangial and endocapillary hypercellularity and crescents were associated with IgA and C3 capillary localization by IF. Mesangial and endocapillary hypercellularity also correlated with IgA and C3 staining intensity by IF, and immunodeposits intensity and degree of FPE by EM. In 115 incident subjects enrolled within 6 months of their kidney biopsy, proteinuria and urinary sC5b9 were associated with IgA and C3 capillary deposition by IF, as well as the quantity of subendothelial deposits by EM. Finally, proteinuria and urinary sC5b9 were each independently associated with a combined outcome and showed an interaction when both were elevated.

**Conclusion:**

In IgAN, the amount and localization of immunodeposits correlated with proliferative lesions and urinary sC5b9. In turn, the urinary membrane attack complex was independently associated with loss of eGFR.

## Graphical abstract

**Figure:**
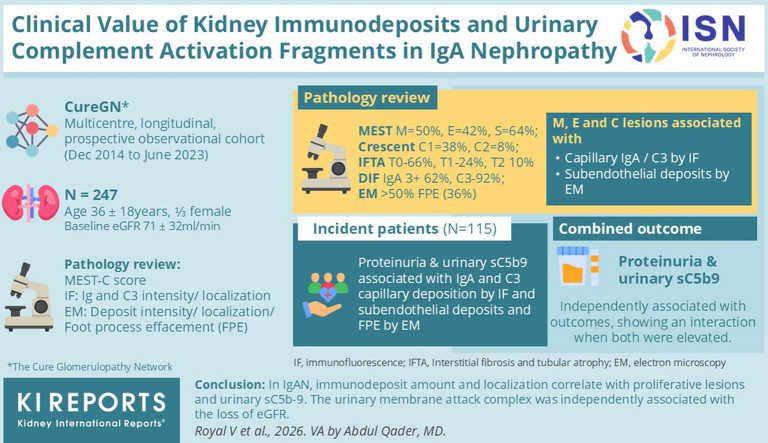

IgAN is the most common primary glomerular disease worldwide and a significant cause of end-stage kidney disease (ESKD).^1^, ^2^, ^3^ The clinical course of IgAN is highly variable, and both treatment strategies and therapeutic responses differ widely among patients. The Oxford classification has provided a standardized framework for evaluating LM features in IgAN, demonstrating both correlations with clinical presentation and independent prognostic value.^4^, ^5^, ^6^ However, despite its broad validation, the Oxford classification offers limited guidance for therapeutic decision-making.^7^ Additionally, it does not incorporate IF and EM findings. As opposed to LM, few studies have explored the prognostic significance of IF findings. These mainly focused on IgG codeposition by IF and did not include urinary marker of complement activation or account for the level of proteinuria in predicting outcomes.^8^, ^9^, ^10^, ^11^, ^12^

Recent studies have characterized IgAN as an autoimmune disease with a multihit pathogenesis. In this model, galactose-deficient IgA1 is produced in elevated amounts and recognized by unique antiglycan autoantibodies, leading to the formation of immune complexes in the circulation and deposition within the mesangium.^13^^,^^14^ Increasing evidence supports a central role for immune complex formation in triggering inflammation and glomerular injury.^15^^,^^16^ Furthermore, complement activation has emerged as a key mediator of glomerular damage in IgAN, primarily through the lectin and alternative pathways.^17^ Moreover, recent studies investigating complement inhibitors for the treatment of IgAN have yielded promising results,^18^, ^19^, ^20^ reinforcing the importance of complement activation. Nevertheless, the relationships between complement activation assessed by IF and EM or by urinary activation fragments, and disease activity and progression are uncertain. It thus also remains unclear whether these parameters can inform therapeutic decision-making.

To address these gaps, we conducted a study using the CureGN IgA cohort. First, we assessed the correlations between LM, IF, and EM findings and clinical presentation, as well as long-term outcomes. Second, we examined associations between urinary sC5b9 and kidney biopsy features, clinical presentation, and long-term outcomes. We hypothesized that the assessment of immunodeposits by IF and EM and urinary complement sC5b9 offer a predictive value additional to the classical risk assessment using LM findings and proteinuria.

## Methods

### Study Design and Population

CureGN (https://curegn.org/) is a multicenter National Institute of Diabetes and Digestive and Kidney Diseases-funded, longitudinal, prospective observation cohort study which enrolled children (< 18 years old at the time of biopsy) and adults with a diagnostic biopsy within the past 5 years with either IgAN / IgA vasculitis, membranous nephropathy, minimal change disease, or focal segmental glomerulosclerosis.^21^ The CureGN network study employs a structured approach to patient and public engagement by incorporating patient-reported outcomes and prioritizing the patient perspective. It collects patient-reported outcomes using validated instruments which allows for quantitative assessment. This approach ensures that the lived experience of patients with glomerular disease is captured and integrated into research analyses and outcome measures. Institutional review board approval was obtained for all participants at their respective enrolling sites. All adult participants and legal guardians of minor participants provided written informed consent.

Institutionalized patients or those with ESKD at screening, diabetes, a previous organ/hematopoietic transplant, hepatitis B or C, HIV, malignancy, illicit drug use, or systemic lupus erythematosus at the time of biopsy were excluded. The CureGN pathology inclusion criteria for IgAN required ≥ 5 glomeruli available for evaluation, and dominant or codominant mesangial IgA staining (≥ 1+) by IF. In this study, all participants with a diagnosis of IgAN / IgA vasculitis, who had their biopsy reviewed by CureGN pathologists from December 2014 to June 2023 were included. There were no eGFR and proteinuria entry criteria.

### Histopathological Evaluation

Central pathology review data were obtained from CureGN, including glomerular, tubulointerstitial, and vascular assessment. Using LM whole-slide images, IF data and, when available, EM images, a central pathology review was performed by the CureGN core scoring working group, who established a standardized approach for scoring of kidney pathology features based on routine clinical diagnostic practices, as previously published.^22^ The Oxford MEST-C (mesangial hypercellularity, endocapillary hypercellularity, segmental glomerulosclerosis, tubular atrophy, and crescents) score was derived using these assessments.^23^

According to the CureGN core scoring protocol, mesangial hypercellularity was scored as absent (M0), focal (< 50% of glomeruli, also M0), or diffuse (≥ 50%, M1). E1 was defined by any glomeruli showing endocapillary hypercellularity. The proportion of crescents was categorized as C0: none; C1: 1% to 25%; and C2: > 25%. This included cellular and fibrocellular whereas excluding fibrous crescents, as in the Oxford classification. The proportion of glomeruli showing segmental sclerosis (S0: none; S1: any) was reported. Interstitial fibrosis and tubular atrophy (IFTA) were graded using a semiquantitative scale from 0 to 3 as follows: 0, normal; 1 (mild), 1% to 25%; 2 (moderate), 26% to 50%; and 3 (severe), > 50% of the cortex. Arterial intimal fibrosis and arteriolar hyalinosis were scored using a semiquantitative scale from 0 to 3 (none, mild, moderate, and severe). CureGN core scoring pathologists extracted IF findings (IgA, C3, IgG, and IgM) from pathology reports, including the intensity of the staining, using a semiquantitative scale from 0 to 3 (negative, mild, moderate, and strong) and, when present, localization (mesangial or mesangiocapillary). In cases where intermediate scores were given, we simplified to the lower end (e.g., 1.5+ simplified to 1+).

Provided EM findings included a semiquantitative assessment of dense deposits found in the mesangium and along the capillary wall, graded as follows: 0 (none), 1+ (few and small), 2+ (numerous and small, or few and large), or 3+ (numerous and large). An assessment of the degree of FPE was provided, using the following semiquantitative score: 0, none; 1 (mild), 1% to 25%; 2 (moderate), 26% to 50%; and 3 (severe), > 50% of surface area of glomeruli.

### Clinical Dataset, Urinary Measurements and Clinical Outcomes

Demographic, laboratory, clinical, and medication variables of patients with IgAN were extracted from the CureGN database and included biological sex, race, ethnicity, age at enrollment, and time from biopsy to enrollment. Baseline eGFR was calculated using the Chronic Kidney Disease in Children Under 25 formula if age < 25 years^24^ or the Chronic Kidney Disease–Epidemiology Collaboration 2021 formula.^25^ We also report at enrollment the blood pressure, use of renin angiotensin aldosterone system blockade, the number of blood pressure medications, and hematuria (coded as trace, 1+, 2+, and 3+ corresponding to 5–10, 11–25, 26–50, and > 50 red blood cells per high power field, respectively). However, these values were not standardized by a central laboratory. Immunosuppressive medications (IS) from the time of biopsy to the end of follow-up were also obtained.

Terminal pathway complement activation was measured in all patients using the human enzyme immunoassay kits for urinary sC5b9, the soluble membrane attack complex, which can be measured in the urine or blood (MicroVue; Quidel Corp., San Diego, CA). Urine samples were diluted 1:3. The lower threshold sensitivity of the assays is 15 ng/ml. Two hundred fifty microliters of centrifuged and stored urine supernatant were collected at enrollment. Urinary protein and creatinine levels were measured from the same sample. Urinary levels of sC5b9 and proteinuria are expressed as a creatinine ratio. Measurements were performed at the Ste-Justine Hospital laboratory in Montréal, Canada.

A composite outcome was defined by the patient meeting at least 1 of the following criteria: (i) ESKD defined as 2 consecutive eGFR < 15 ml/min per 1.73 m^2^ more than 1 month apart, or (ii) eGFR decline ≥ 40% based on decline from enrollment using regression method among patients with a completed enrollment visit.

### Statistical Analyses

The analyses were reported in the following order: (i) associations between LM with IF and EM findings; (ii) associations between LM and urinary measurements (proteinuria and sC5b9/creatinine ratio); (iii) associations between IF and EM with urinary findings; (iv) associations between LM, IF, and EM findings with clinical outcomes; and (v) associations between urinary measurements and clinical outcomes. Given the variable time between biopsy and urinary measurements, we limited the analyses between pathology and urinary measurements to incident patients, enrolled within 6 months of pathology.

Normally distributed variables are presented as means ± SD and compared using 1-way ANOVA. Nonparametric variables are expressed as medians with interquartile range and compared using the Kruskal-Wallis test. Correlations and trend tests between 3 or more ordinal groups with nonparametric measurements were done using Spearman’s ρ. Categorical variables are summarized using proportions and compared using the Pearson chi-square test.

The time-to-event for urinary biomarkers was calculated from the measurement at enrollment to the last follow-up or the combined outcome. The time-to-event for pathology findings was calculated from the time of biopsy. We analyzed the associations between pathology findings and a combined event using Kaplan-Meier curves and the log-rank test. Since clinical assessment at the time of biopsy (proteinuria, eGFR) was unavailable, we did not perform multivariate analysis using pathology findings. The relation between proteinuria and urinary sC5b9 with a combined event was tested using Cox proportional hazards regression. We adjusted these findings for clinical variables available (age, sex, and eGFR at enrollment). We did not replace missing data. We also examined whether an interaction existed between the 2 urinary measurements. To illustrate these findings with Kaplan-Meier curves, we categorized urinary measurements into groups. Statistical analyses were performed using SPSS for Windows (SPSS version 21.0, IBM Corp., Armonk, NY). All analyses were 2-sided; a *P* value of 0.05 or less was considered statistically significant. We also report hazard ratios and 95% confidence intervals.

## Results

### Cohort Characteristics

There were 247 CureGN patients with IgAN and biopsy review. At enrollment, the age was 36 ± 18 years, with an eGFR of 71 ± 32 ml/min per 1.73 m^2^ and one-third of the participants were female (Table 1). Hypertension was reported by 52% of subjects; other comorbidities were few— 8 (3.2%) had diabetes, 8 (3.2%) had a history of cardiovascular disease, and 4 (1.6%) had a history of heart failure. The median body mass index was 26 (23–31).

**Table 1:** Baseline characteristics of 247 CureGN IgA with biopsy review

Variable	
At biopsy	
Age (yrs)	35 ± 18
Female (%)	34
Race (% Asian, African, Caucasian, Other/unknown)	11, 4, 79, 6
Immunosuppression from biopsy to enrollment, n (%)	146 (59)
At enrollment	
History of Henoch-Schönlein purpura, *n* (%)	53 (21)
Age at inclusion (yrs)	36 ± 18
Age < 18, *n* (%)	54 (22)
Time from biopsy to inclusion (mo)	4.4 (1.3–23.3)
eGFR (ml/min per 1.73 m^2^)	71 ± 32
Blood pressure (mm Hg)	122 ± 17 / 76 ± 12
Renin angiotensin aldosterone blockade, *n* (%)	172 (70)
Number of anti-hypertensive drugs	1 (1–2)
Urinary protein to creatinine ratio (g/g of creatinine)	0.77 (0.18–2.06)
Urinary sC5b9 to creatinine ratio (μg/mmol creatinine)	0.22 (0.00–1.47)
Urinary red blood cells (% absent, trace, 1+, 2+, 3+)	18, 8, 14, 24, 36
Follow-up	
Time follow-up from enrollment (yrs)	6.2 (4.9–7.0)
Immunosuppression after enrollment (*n*, %)	124 (50)
Time of start of prospective immunosuppression (mo)	0 (0–3)
≥ 40% decline in eGFR, *n* (%)	64 (26)
End-stage kidney disease, *n* (%)	46 (19)
Combined event, *n* (%)	73 (30)

CureGN, Cure Glomerulonephropathy; eGFR, estimated glomerular filtration rate.

Hematuria was coded as trace, 1+, 2+, and 3+ corresponding to 5–10, 11–25, 26–50 and >50 red blood cells per high-power field, respectively.

The proteinuria was 0.77 (0.18–2.06) g/g of creatinine and the blood pressure 122/76 mm Hg, with 70% receiving renin angiotensin aldosterone system blockade, and two-thirds of the subjects had significant hematuria. The initial urinary sC5b9 to creatinine ratio was 0.22 (0.00–1.47) μg/mmol of creatinine; it did not correlate with the eGFR (Spearman’s ρ = −0.11, *P* = 0.10). One hundred and fifteen patients had their biopsy within 6 months of their enrollment and were considered incident. Compared with prevalent patients, incident subjects at enrollment were older (38 ± 18 vs. 34 ± 17, *P* = 0.02) with a higher proteinuria (1.1, 0.4–2.6 vs. 0.4, 0.0–1.5, *P* < 0.001) but similar initial eGFR (72 ± 32 vs. 69 ± 31 ml/min per 1.73 m^2^). Fifty-three subjects (21%) had a history of Henoch-Schönlein purpura at enrollment.

Fifty-nine percent had received IS in the 4.4 months (1.3–23.3) from the biopsy to enrollment. This included the use of antimetabolites in 5 (2.0%), corticosteroids in 141 (57%), cyclophosphamide in 14 (5.7%), calcineurin inhibitors in 7 (2.8%), mycophenolate in 26 (11%) and anti-B cell therapy in 2 (0.8%). Fifty percent received IS thereafter as follows: ACTH was used in 2 (0.8%) subjects, antimetabolites in 22 (8.9%), corticosteroids in 118 (48%), cyclophosphamide in 11 (4.5%), calcineurin inhibitors in 35 (14.2%), mycophenolate in 48 (19%) and anti-B cell therapy in 7 (2.8%). Patients were followed for a median of 6.2 (4.9–7.0) years, during which 73 (30%) experienced a combined event (64 had a ≥ 40% decline in eGFR and 46 reached ESKD).

There was little missing clinical information. Only 1 patient had no urinary protein and sC5b9 measurements. The initial eGFR was unavailable for 23 patients. A ≥ 40% decline in kidney function was then calculated from the first available eGFR. Blood pressure, body mass index, and hematuria were missing in 14, 3, and 40 subjects, respectively.

### Pathology Review

**Figure 1:**
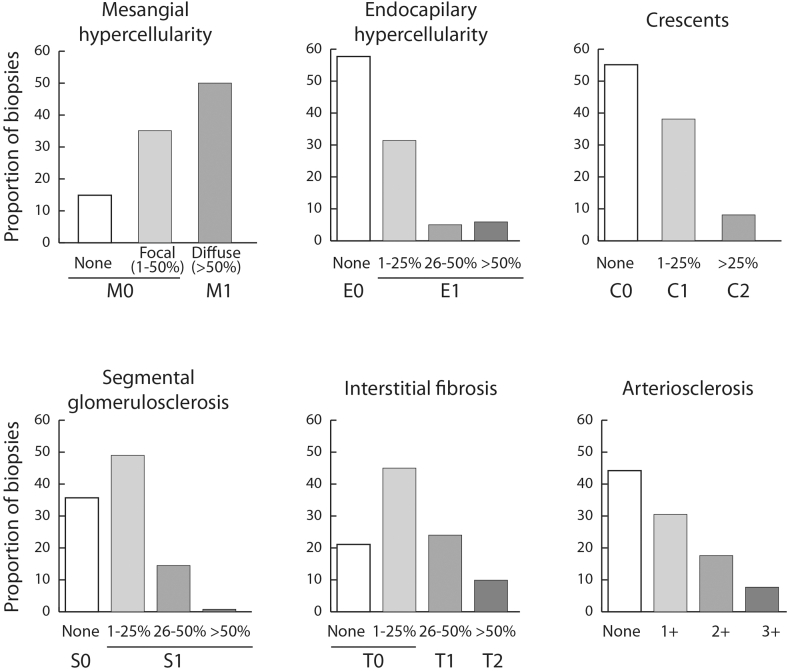
Light microscopy findings. The MEST-C score was extrapolated from the CureGN assessment. Fibrous crescents were included in CureGN as opposed to the original Oxford score, which only included cellular and fibrocellular crescents.

LM findings are shown in Figure 1. With a median of 17 (12–27) glomeruli, the mesangial hypercellularity (M), endocapillary hypercellularity (E), and segmental glomerulosclerosis (S) lesions were found in 50%, 42%, and 64% of cases, respectively. Crescents were categorized as absent in 54%, C1 in 38%, and C2 in 8%. IFTA was scored as T0 in 66%, T1 in 24%, and T2 in 10%.

**Figure 2:**
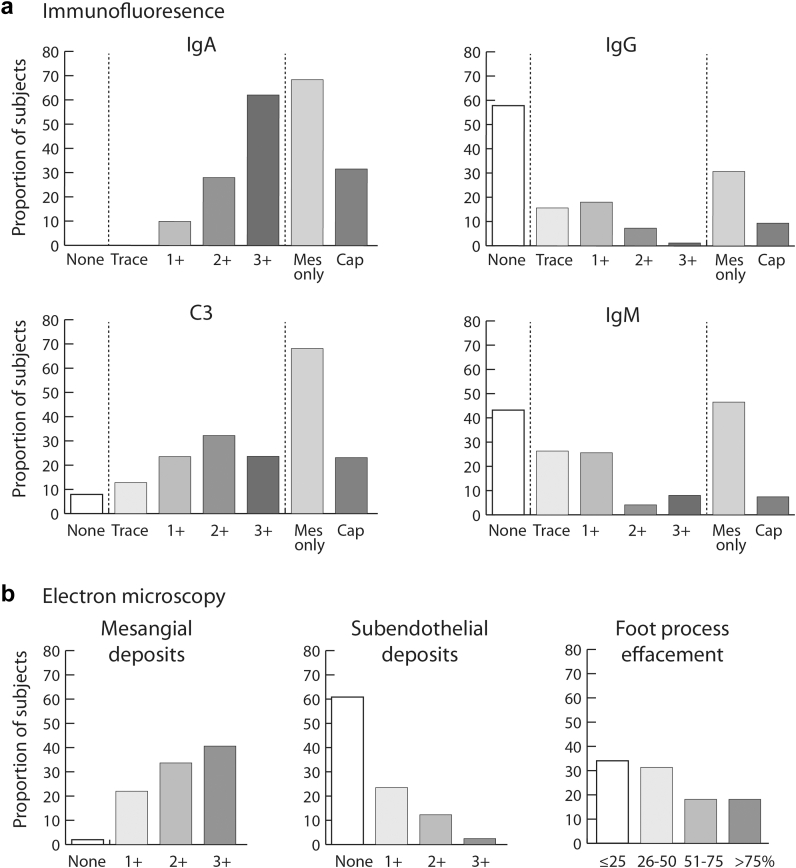
Immunofluorescence (a) and electron microscopy (b) findings. Mes, mesangial; Cap, capillary.

Findings by IF and EM are shown in Figure 2. By definition, all patients had at least 1+ IgA deposits, with 62% having 3+. Thirty percent of biopsies showed IgA deposits in the capillary walls in addition to the mesangium. C3 was detected in 92% of biopsies, with a grade of ≥ 2+ in 56%, and was deposited in capillaries in 25% of cases. IgG and IgM deposits were not abundant and rarely found in the capillaries. By EM, only 2% had no mesangial deposits, whereas 62% had no subendothelial deposits. FPE was frequent, with 36% of cases showing greater than 50% effacement.

LM and IF data were missing in 3% or less. EM findings were missing in 7% of cases.

### Associations Between LM With IF and EM Findings

**Figure 3:**
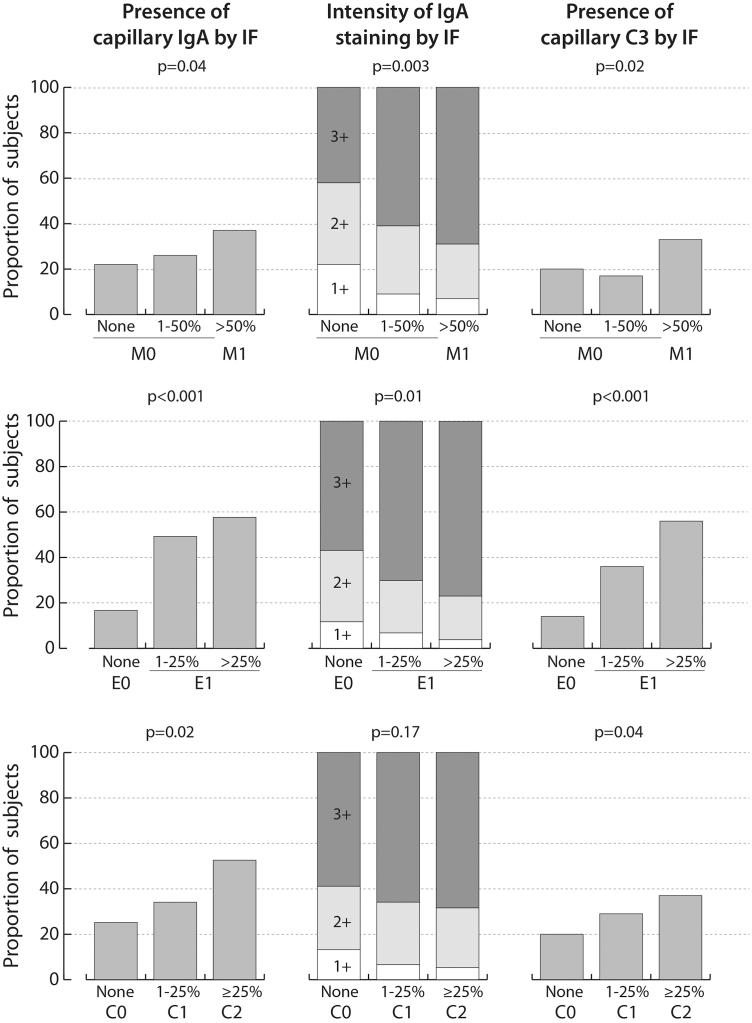
Associations between light microscopy and immunofluorescence. Segmental sclerosis also correlated with the intensity and localization of IgM staining. Endocapillary hypercellularity was associated with the intensity and localization of IgM staining (data not shown). Trend tests were performed using Spearman’s ρ. dep, deposition; IF, immunofluorescence.

Crescents, mesangial, and endocapillary hypercellularity correlated with the presence of IgA and C3 in the capillaries (Figure 3). Mesangial hypercellularity was marginally associated with C3 staining intensity (Spearman’s ρ = 0.13, *P* = 0.04). Mesangial and endocapillary hypercellularity were also related to the IgA staining intensity and the quantity of mesangial and subendothelial deposits by EM (Figure 3 and Supplementary Figure S1), whereas crescents correlated with subendothelial deposits by EM. Segmental sclerosis only correlated with the intensity and capillary localization of IgM staining (data not shown). Finally, endocapillary hypercellularity was associated with the intensity and localization of IgG staining (Supplementary Figure S2). Additional associations between IF and EM findings are listed in Supplementary Table S1.

### Associations Between LM and Urinary Measurements

**Figure 4:**
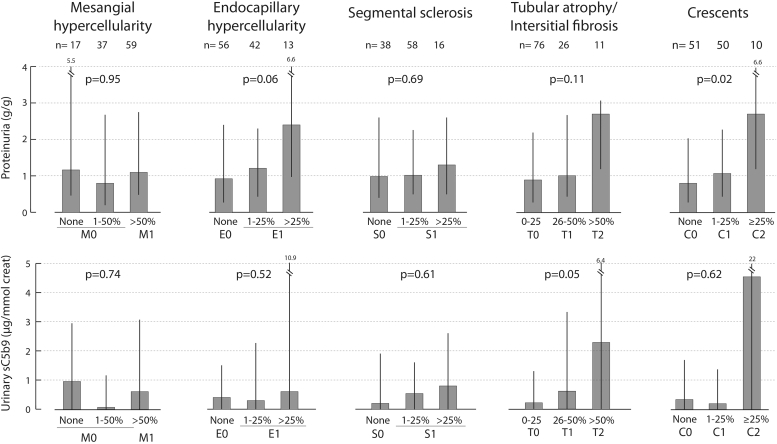
Association between light microscopy findings and proteinuria and urinary sC5b9 at enrollment in incident patients (*n* = 115). Incident patients were defined by ≤ 6 months between biopsy and enrollment. Trend tests were performed using Spearman’s ρ.

Given the variable time between biopsy and urinary measurements, we limited these analyses to the 115 incident subjects. Still, in these incident patients, 55% had been exposed to IS therapy before enrollment and urinary sampling. The associations between LM findings and urinary biomarkers were poor (Figure 4). Although trends existed, only crescents were associated with proteinuria, whereas urinary sC5b9 correlated with IFTA.

### Associations Between IF and EM With Urinary Measurements

**Figure 5:**
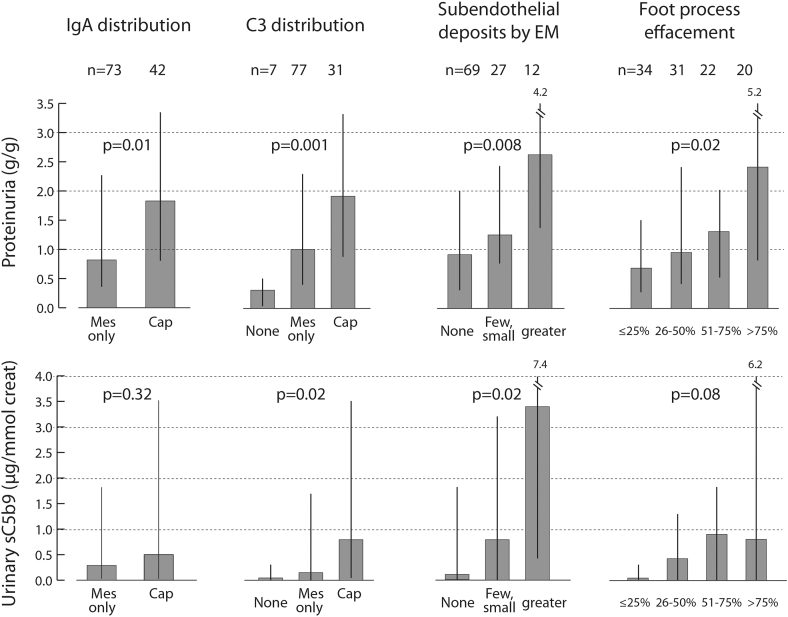
Associations between immunofluorescence and electron microscopy findings with urinary measurements in incident patients (*n* = 115). EM, electron microscopy. Incident patients were defined by ≤ 6 months between biopsy and enrollment. Trend tests were performed using Spearman’s ρ.

In the same subset of incident patients, proteinuria was associated with the presence of capillary IgA and C3, EM subendothelial deposits, and FPE (Figure 5). Urinary sC5b9 was also related to capillary C3 and EM subendothelial deposits. There were no significant associations between the intensity of any immunodeposits found by IF and urinary measurements (data not shown).

### Associations Between Pathology Findings and Clinical Outcomes

Endocapillary hypercellularity and crescents were associated with the subsequent use of IS, whereas mesangial hypercellularity, segmental sclerosis, and IFTA were not (Supplementary Table S2). There were no IF findings associated with the use of IS. However, a higher intensity of subendothelial deposits and a higher proportion of FPE were associated with greater IS treatment.

Only segmental sclerosis and IFTA, as well as the degree of FPE, were predictive of survival from a ≥ 40% decline in renal function or ESKD (Supplementary Figure S3). No other LM, IF, or EM findings were associated with a combined outcome. Since the eGFR and proteinuria at the time of biopsy were not available, we could not evaluate their independent value.

### Associations Between Urinary Findings and Clinical Outcomes

Higher proteinuria was associated with greater IS after enrollment. Seventy-four percent of subjects with the highest tertile of proteinuria received IS, as opposed to 26% in the lowest tertile. Similarly, 64% of those in the highest urinary sC5b9 tertile received IS, compared with 43% in the lowest tertile (Supplementary Table S3). There was no association between sex, initial eGFR, and age with the level of urinary biomarkers.

**Figure 6:**
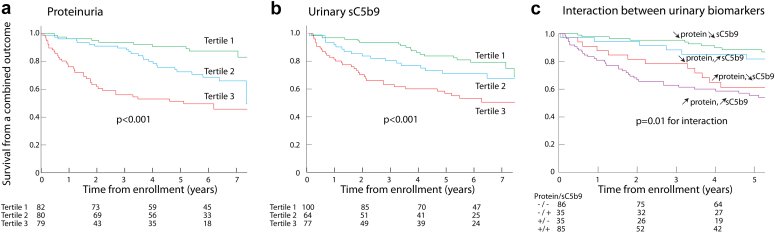
Survival from a combined event according to tertiles of (a) proteinuira, (b) urinary sC5b9, and (c) their interaction. Time is calculated from enrollment to event or end of follow-up. An interaction term was obtained from the Cox regression model in Table 2.

Higher urinary biomarkers were strongly associated with survival from a combined outcome (Figure 6a and b). Since proteinuria and urinary sC5b9 (a protein) showed a very high correlation (Spearman’s ρ: 0.64, *P* < 0.001), we performed a multivariate Cox regression to assess each urinary measurement’s independent value, adjusted for sex, initial age, and eGFR (Table 2). We also included an interaction term to examine the interdependence between the 2 biomarkers (Table 2). Proteinuria and urinary sC5b9 at enrollment were independently associated with survival from a combined event, and their effects showed an interaction. To illustrate this, when dichotomized into low and high groups, we observe that a high urinary sC5b9 increased the risk of an event only in the high-proteinuria group (Figure 6c).

**Table 2:** Independent associations between proteinuria and urinary sC5b9 with survival from a combined outcome

Variable at enrollment	Hazard ratio	95% Confidence interval	*P-*value
Age (10-yr increment)	0.85	0.70–1.02	0.08
Sex (female patient)	1.41	0.86–2.30	0.18
eGFR (10ml/min per 1.73 m^2^ increment)	0.66	0.58–0.75	< 0.001
Proteinuria (g/g of creatinine)	1.35	1.17–1.56	< 0.001
Urinary sC5b9 (μg/mmol of creatinine)	1.16	1.05–1.29	0.004
Proteinuria and urinary sC5b9 interaction	0.98	0.97–0.996	0.01

eGFR, estimated glomerular filtration rate; sC5b9, soluble C5b9.

## Discussion

This study found that in IgAN, immunodeposits found by IF and EM correlated with disease activity as determined by LM findings. The presence of capillary IgA and C3 and the severity of EM subendothelial deposits and FPE were associated with greater proteinuria. In addition, capillary C3 and EM subendothelial deposits also correlated with urinary sC5b9. Finally, despite receiving greater IS, subjects with higher levels of proteinuria and urinary sC5b9 experienced a poorer kidney outcome, independent of each other.

The strong association between the capillary localization of immunodeposits and urinary sC5b9, in addition to hypercellularity by LM, and, in turn, the association found between urinary sC5b9 and kidney outcomes are novel. Since its initial description by Berger, other investigators have explored the clinical significance of IF staining patterns in IgAN. Our findings are consistent with previous studies, which demonstrated a correlation between the localization of immunodeposits and disease activity as observed by LM^8^ and earlier ultrastructural findings by EM.^26^ The subendothelial localization of immunodeposits may reflect an overburden of immune complex or an alteration in the complex size, charge, or composition. These changes could allow immune complex to localize beneath the endothelial layer, where they may trigger an endocapillary inflammatory response. This, in turn, can promote complement activation, leukocyte recruitment, leading to endocapillary hypercellularity. Supporting this, we observed that capillary wall C3 staining by IF correlated with higher levels of urinary sC5b9, underscoring the role of complement activation in the pathogenesis of IgAN. As opposed to mesangial deposition, capillary sC5b9 could also facilitate its passage into the urine.

Few studies have described associations between IF staining and disease progression, mainly linking IgG, C3, and/or C4d mesangial deposition with worse kidney outcomes.^27^, ^28^, ^29^ In a retrospective study of 98 children with biopsy-proven IgAN, Wu *et al.*^30^ found that mesangial deposition of C3 ≥ 2+ was significantly associated with a higher risk of a 30% decline in GFR or kidney failure. In this study, there were no significant associations between the IF or EM findings and kidney outcomes, except for the degree of FPE, although proliferative lesions were associated with a greater use of IS, which may have lessened their predictive value. Moreover, findings from our study support the contribution of other Ig isotypes in the pathogenesis of IgAN. Suzuki *et al.*^14^ reported elevated glycan-specific IgG antibody levels in patients with IgAN with mesangial deposition, which were shown to induce a mesangioproliferative glomerular injury. We observed that the intensity of IgG staining, as well as a capillary localization, were significantly associated with active glomerular lesions. Others have also found a potential pathogenic role for IgG in amplifying the inflammatory response in IgAN.^8^ Perhaps the IgG staining also reflects the burden of immunocomplexes, which then allows the detection of IgG by routine IF.^13^

Despite significant advances in our understanding of the physiopathology of IgAN, there remains a need for noninvasive biomarkers in IgA nephropathy to better define individual risk profiles and inform therapeutic decisions. Complement activation has emerged as a central mechanism in the pathophysiology of IgAN, particularly through activation of the alternative and lectin pathways,^31^, ^32^, ^33^ and the recent therapeutic advances with complement inhibition further highlight its importance. In Phase II and III trials, the alternative pathway inhibitor Iptacopan significantly reduced proteinuria and had a favorable safety profile.^18^^,^^20^ Similarly, the C5 inhibitor Ravulizumab also demonstrated a reduction in proteinuria with good tolerability, supporting complement blockade as a promising treatment strategy in IgAN.

Emerging data suggest that complement activation within the kidney causes direct tissue injury.^34^, ^35^, ^36^ In addition to being easily accessible, urinary complement peptides may better reflect intrarenal inflammation, as opposed to serum biomarkers, likely influenced by systemic inflammation. Several studies have addressed the potential role of urinary complement proteins, from both lectin and alternative pathways, as potential biomarkers of activity and chronicity in IgAN. Some associations have been described between urinary markers and histological activity, clinical presentation, and kidney outcomes, showing variable yet promising results.^37^^,^^38^ In a study of 100 patients with IgAN, Wang *et al.*^39^ showed that urinary levels of C3a, C5a, C4d and mannose-binding lectin, and also C5b9 correlated with the proportion of glomerular crescents, serum creatinine levels, and proteinuria. Another study of 508 patients with biopsy-proven IgAN demonstrated that higher levels of urinary C4d-to-creatinine ratio were associated with lower eGFR, higher proteinuria, endocapillary hypercellularity, tubular atrophy, and increased chronic kidney disease progression, although the correlation with immunodeposits was not reported.^40^ In a recent Swedish cohort of 96 patients with IgAN and IgA vasculitis, Nurmi *et al.*^41^ found higher Pentraxin 3 and mannose-binding lectin in patients with more histologically active disease, and higher urinary C4c in those with interstitial fibrosis and more severe eGFR slope.^41^ Also, patients with detectable urinary sC5b9 had a greater extent of IFTA, suggesting that tubule-interstitial injury could be a source of urinary sC5b9. Interestingly, animal models have linked C5b9 deposition to the development of progressive tubulo-interstitial damage.^42^^,^^43^ Similar findings have also been demonstrated in lupus nephritis.^44^ In this study, although trends existed between urinary sC5b9 and E and C scores, only the degree of IFTA and the extent of FPE were associated with urinary sC5b9.

Additionally, our findings highlight the plausible role of podocyte injury in the progression of IgAN. Both the extent of FPE and the presence of segmental sclerosis were associated with the composite outcome. Furthermore, FPE correlated not only with proteinuria but also with urinary levels of C5b9, suggesting a link between podocyte injury and complement activation. Podocyte damage has been increasingly recognized as a central contributor to glomerular injury and a predictor of kidney outcomes in IgAN.^45^ Mechanistically, C5b9 has been shown to induce podocyte injury mainly through sublytic injury, leading to DNA damage and inability to proliferate.^46^^,^^47^

Some limitations deserve comments. The median proteinuria at enrollment was only 0.77 g/g of creatinine, lower compared with other observational studies, such as the Oxford and VALIGA cohorts. Additionally, there was a 4.4-month difference between the kidney biopsy and the first available clinical assessment, unlike other studies. This difference reached 2 years in about a quarter of patients, inevitably influencing any clinicopathologic correlations. To address this, we limited these analyses to incident patients defined by a ≤ 6-month period from biopsy to clinical assessment; however, even within this group, 55% of subjects had already been exposed to immunosuppression, which can reduce proteinuria and most likely complement activation. Limiting our analyses to incident patients who had not received immunosuppression before enrollment reduced our cohort to 52, reducing our statistical power. This, in turn, may explain the disappointing correlations between LM and proteinuria. This finding does not invalidate previous studies; rather, it reflects our lack of power because of the limitations cited above. However, despite poor correlations between LM and urinary biomarkers, we still found that urinary sC5b9 was associated with capillary C3 and EM subendothelial deposits. Another limitation is that urinary biomarkers were measured only once, and the significant use of IS afterwards may impact their predictive value. A greater predictive value could have been found with repeated measurements. Predictably, we found greater IS with higher levels of urinary biomarkers, but despite this, we still demonstrate an independently higher risk of progression with higher urinary sC5b9 levels.

In conclusion, the findings from the CureGN cohort presented here strongly support the consideration of immunodeposits assessed by IF and EM in IgAN. At the same time, the limitations cited above preclude their inclusion to the MEST-C score at this time, but future studies should assess this, particularly in those who do not receive immunosuppression. We propose a simple urinary biomarker of complement activation to assess the risk of progression. In turn, this may facilitate the assessment of new therapies that precisely suppress the production of Igs or inhibit their ability to trigger the complement cascade.

## Disclosure

AB reports consultancy from Alexion Pharmaceuticals, Sanofi, and BioCryst; honoraria at Alexion Pharmaceuticals, Sanofi, BioCryst; and advisory or leadership role at Alexion Pharmaceuticals, Sanofi, and BioCryst.

YC reports consultancy at Vera Therapeutics; and scientific advisor for Nephronomics.

L-PL reports consultancy from Otsuka Pharmaceutical, and Novartis; and advisory or leadership role at Otsuka Pharmaceutical.

KL reports consultancy at CareDx, Inc., and Maze Therapeutics; speakers bureau at Sanofi; and author at UpToDate.

VR reports the honoraria at Novartis, Otsuka Pharmaceutical, and Sobi.

CM and ST declared no competing interests.

## Appendix

**CureGN Participating Clinical Centers (PCC) through Columbia University:**

*Columbia University, New York, NY, US*: Gerald Appel, Revekka Babayev, Ibrahim Batal^+^, Andrew Bomback^∗∗^, Pietro Canetta, Brenda Chan, Vivette Denise D'Agati^+^, Samitri Dogra, Hilda Fernandez, Gabriele Gaggero^+^, Ali Gharavi^∗∗^, William Hines, Krzysztof Kiryluk^∗∗^, Satoru Kudose^+^, Fangming Lin, Victoria Kolupaeva#, Maddalena Marasa, Glen Markowitz^+^, Mariela Navarro-Torres, Hila Milo Rasouly, Sumit Mohan, Nicola Mongera, Jordan Nestor, Jai Radhakrishnan, Maya Rao, Maya Sabatello, Simone Sanna-Cherchi, Dominick Santoriello^+^, Miroslav Sekulic^+^, Michael Barry Stokes^+^, Natalie Uy, Natalie Vena, Benjamin Wooden

*University of Warsaw, Warszawa, Poland:* Bartosz Foroncewicz, Natalia Wiewiórska-Krata, Barbara Moszczuk, Krzysztof Mucha^∗^, Agnieszka Perkowska-Ptasińska, Elżbieta Ryszkowska

*IRCCS Giannina Gaslini, Genoa, Italy*: Francesca Lugani, Valerio Vellone^+^

**CureGN Participating Clinical Centers (PCC) through the Pediatric Nephrology Research Consortium:**

*Children’s Hospital of New Orleans/ LSU Health, New Orleans, LA, USA*: Diego Aviles∗

*Children’s Mercy Hospital, Kansas City, MO, USA*: Tarak Srivastava^∗^, Alexander Katz^+^

*Children’s National Medical Center, Washington DC, USA*: Sun-Young Ahn∗

*Cincinnati Children’s Hospital Cincinnati, OH, USA*: Prasad Devarajan, Elif Erkan^∗^, Hillarey Stone

*Connecticut Children’s Medical Center, Hartford, CT, USA*: Sherene Mason∗

*East Carolina University Brody School of Medicine, Greenville, NC, USA*: Liliana Gomez-Mendez∗

*Emory University, Atlanta, GA, USA*: Larry Greenbaum^∗∗^, Chia-shi Wang, Hong (Julie) Yin^+^

*Helen DeVos Children’s Hospital, Grand Rapids, MI, USA*: Jens Goebel∗

*Levine Children’s Hospital/Atrium Health, Charlotte, NC, USA*: Donald Weaver∗

*Lurie Children’s Hospital, Chicago IL, USA*: Jill Krissberg^∗^, Jerome Lane

*Medical College of Wisconsin, Milwaukee, WI, USA*: Cindy Pan, Ellen Cody∗

*Nationwide Children’s Hospital, Columbus, OH, USA*: Samantha Martinek-Bundt^#^, Dawson Carmean^#^, Mary Dreher^#^, Mahmoud Kallash^∗^, John Mahan^∗∗^, Samantha Sharpe^#^, William Smoyer^∗∗^, Laura Biederman^+^

*Oregon Health and Science University, Portland, OR, USA*: Chloe Douglas^∗^, Sandra Iragorri

*Riley Children’s Hospital, Indianapolis, IN, USA*: Myda Khalid^∗∗^

*Cardinal Glennon Children’s Medical Center/ St. Louis University, St. Louis, MO, USA*: Craig Belsha∗

*Texas Children’s Hospital, Houston, TX, USA*: Elizabeth Onugha^∗^, Michael Braun, AC Gomez

*Texas Tech Health Sciences Center, Amarillo, TX, USA*: Tetyana Vasylyeva∗

*Children’s of Alabama, University of Alabama, Birmingham, AL, USA*: Daniel Feig∗

*University of Colorado Children’s Hospital, Colorado, Aurora, CO, USA*: Melisha Hannah∗

*University of Kentucky, Lexington, KY, USA*: Aftab Chishti∗

*University of Louisville, Louisville, KY, USA*: Jon Klein^∗∗^

*Holtz Medical Center, University of Miami, Miami, FL, USA*: Chryso Katsoufis, Wacharee Seeherunvong∗

*University of Minnesota Children’s Hospital, Minneapolis, MN, USA*: Michelle Rheault^∗∗^

*University of New Mexico Health Sciences Center, Albuquerque, NM, USA*: Craig Wong∗

*University of Oklahoma Health Sciences Center, Oklahoma City, OK, USA*: Qassim Abid∗

*University of Virginia, Charlottesville, VA, USA*: John Barcia^∗^, Agnes Swiatecka-Urban

*University of Wisconsin, Madison, WI, USA*: Sharon Bartosh∗

*Washington University in St. Louis, St. Louis, MO, USA*: Brian Stotter^∗^, Joseph Gaut^+^

**CureGN Participating Clinical Centers (PCC) through the University of North Carolina:**

*Hôpital Maisonneuve-Rosemont, Montreal, Canada*: Louis-Philippe Laurin^∗^, Virginie Royal^+^, Mathieu Latour^+^, Natlie (Natacha) Patey^+^

*Medical University of South Carolina, Charleston, SC, USA*: Anand Achanti, Milos Budisavljevic^∗^, Vishwajeeth Pasham^+^

*Northwestern University, Chicago, IL, USA*: Cybele Ghossein, Yonatan Peleg∗

*Ohio State University, Columbus, OH, USA*: Isabelle Ayoub^∗^, Samir Parikh, Brad Rovin, Anjali Satoskar^+^

*University of Chicago, Chicago, IL, USA*: Anthony Chang^+^

*University of Alabama at Birmingham, Birmingham, AL, USA*: Huma Fatima^+^, Jan Novak, Matthew Renfrow, Dana Rizk∗

*University of North Carolina Kidney Center, Chapel Hill, NC, USA*: Dhruti Chen, Vimal Derebail^∗∗^, Ronald Falk^∗∗^, Keisha Gibson, Dorey Glenn, Susan Hogan, Koyal Jain, J. Charles Jennette^+^, Vanessa Moreno^+^, Amy Mottl, Caroline Poulton^#^, Monica Reynolds, Manish Kanti Saha, Nicole E. Wyatt

*Vanderbilt University, Nashville, TN, USA*: Agnes Fogo^+^, Neil Sanghani∗

*Virginia Commonwealth University, Richmond, VA, USA*: Jason Kidd^∗^, Selvaraj Muthusamy^+^

**CureGN Collaborators**

^∗∗^CureGN PCC Principal Investigators; ^∗^CureGN Site Principal Investigators; ^+^CureGN Pathologists, ^#^CureGN Lead Coordinators.

**CureGN Participating Clinical Centers (PCC) through the University of Pennsylvania:**

*Children’s Hospital of Philadelphia, Philadelphia, PA, USA*: Rebecca Scobell∗, Michelle Denburg, Amy Kogon, Kevin Meyers, Madhura Pradhan

*Cleveland Clinic, Cleveland, OH, CA*: Raed Bou Matar∗, John O'Toole, John Sedor

Cohen Children’s Medical Center, New Hyde Park, NY, USA: Christine Sethna∗∗, Suzanne Vento^#^

*Johns Hopkins University, Baltimore, MD, USA*: Mohamed Atta, Serena Bagnasco^+^, Alicia Neu, John Sperati∗

*Lundquist Institute at Harbor-UCLA Medical Center, Torrance, CA, USA*: Sharon Adler∗, Tiane Dai, Ram Dukkipati

*Montefiore Medical Center, The Bronx, New York, NY, USA*: Frederick Kaskel, Kaye Brathwaite, Kimberly Reidy∗

*New York University, New York, NY, USA*: Laura Malaga-Dieguez∗

*Spokane Providence Medical Center, Spokane, WA, USA*: Katherine Tuttle∗

*Stanford University, Palo Alto, CA, USA*: Richard Lafayette∗, Kamal Fahmeedah, Elizabeth Talley

*Sunnybrook Health Sciences Centre, Toronto, Canada*: Michelle Hladunewich∗

*The Hospital for Sick Children, Toronto, Canada*: Rulan Parekh∗

*University Health Network, Toronto, Canada*: Carmen Avila-Casado^+^, Daniel Cattran∗, Reich Heather, Meherzad Kutky

*University of Miami, Miami, FL, USA*: Yelena Drexler∗, Alessia Fornoni

*University of Michigan, Ann Arbor, MI, USA*: Jeffrey Hodgin^+^, Andrea Oliverio∗

*University of Pennsylvania, Philadelphia, PA, USA*: Jon Hogan, Lawrence Holzman∗∗, Matthew Palmer^+^, Gaia Coppock

*University of Pittsburgh School of Medicine, Pittsburgh, PA, USA*: Michael Mortiz, Juhi Kumar∗

*University of Washington, Seattle, WA, USA*: Charles Alpers^+^, J. Ashley Jefferson∗

*UT Southwestern, Dallas, TX, USA*: Kamal Sambandam, Bethany Roehm∗

*University of Minnesota*: Patrick Nachman∗

**Data Coordinating Center (DCC):**

*Cedar Sinai Medical Center, Los Angeles, CA, USA*: Cynthia Nast^+^, Jean Hou^+^

*Duke University, Durham, NC, USA*: Laura Barisoni

*Cleveland Clinic, Cleveland, OH, USA:* Crystal Gadegbeku∗∗

*Northwestern University, Chicago, IL, USA:* Abigail Smith∗∗

*University of Michigan, Ann Arbor, MI, USA*: Brenda Gillespie, Bruce Robinson, Matthias Kretzler, Zubin Modi, Laura Mariani∗∗

## Supplementary Material

Supplemental file (PDF) **Figure S1.** Associations between light microscopy and electron microscopy findings. **Figure S2.** Association between endocapillary hypercellularity and IgG intensity and localization by immunofluorescence. **Figure S3.** Univariate associations between pathology and survival from a combined event. **Table S1.** Associations between immunofluorescence and electron microscopy findings. **Table S2.** Associations between pathology findings by light microscopy and the subsequent use of immunosuppressive therapy. **Table S3.** Comparison of clinical features at measurement of tertiles of proteinuria and urinary sC5b9. STROBE checklist.

## Footnotes

Supplementary file (PDF)
**Figure S1.** Associations between light microscopy and electron microscopy findings. **Figure S2.** Association between endocapillary hypercellularity and IgG intensity and localization by immunofluorescence. **Figure S3.** Univariate associations between pathology and survival from a combined event. **Table S1.** Associations between immunofluorescence and electron microscopy findings. **Table S2.** Associations between pathology findings by light microscopy and the subsequent use of immunosuppressive therapy. **Table S3.** Comparison of clinical features at measurement of tertiles of proteinuria and urinary sC5b9. STROBE checklist.

## References

[1] D’Amico G. (1987). The commonest glomerulonephritis in the world: IgA nephropathy. Q J Med.

[2] D’Amico G., Minetti L., Ponticelli C. (1986). Prognostic indicators in idiopathic IgA mesangial nephropathy. Q J Med.

[3] Alamartine E., Sabatier J.C., Guerin C., Berliet J.M., Berthoux F. (1991). Prognostic factors in mesangial IgA glomerulonephritis: an extensive study with univariate and multivariate analyses. Am J Kidney Dis.

[4] Barbour S.J., Coppo R., Zhang H. (2022). Application of the International IgA Nephropathy Prediction Tool one or two years post-biopsy. Kidney Int.

[5] Coppo R., Cook H.T., Working Group of the International Ig ANN (2009). The Oxford classification of IgA nephropathy: rationale, clinicopathological correlations, and classification. Kidney Int.

[6] Cook H.T., Troyanov S., the Renal Pathology S., Working Group of the International Ig ANN (2009). The Oxford classification of IgA nephropathy: pathology definitions, correlations, and reproducibility. Kidney Int.

[7] Kidney Disease: Improving Global Outcomes (KDIGO) Glomerular Diseases Work Group (2021). KDIGO 2021 clinical practice guideline for the management of glomerular diseases. Kidney Int.

[8] Bellur S.S., Troyanov S., Cook H.T., Roberts I.S., Renal Pathology S., Working Group of International Ig ANN (2011). Immunostaining findings in IgA nephropathy: correlation with histology and clinical outcome in the Oxford classification patient cohort. Nephrol Dial Transplant.

[9] Rizk D.V., Novak L., Hall S.D. (2023). Colocalization of IgG and IgA heavy chains with kappa and lambda light chains in glomerular deposits of IgA nephropathy patients using high-resolution confocal microscopy and correlation with Oxford MEST-C scores. J Clin Med.

[10] Shin D.H., Lim B.J., Han I.M. (2016). Glomerular IgG deposition predicts renal outcome in patients with IgA nephropathy. Mod Pathol.

[11] Alvarado A.S., Andeen N.K., Brodsky S. (2018). Location of glomerular immune deposits, not codeposition of Ig G, influences definitive renal outcomes in Ig A nephropathy. Nephrol Dial Transplant.

[12] Katafuchi R., Nagae H., Masutani K., Tsuruya K., Mitsuiki K. (2019). Comprehensive evaluation of the significance of immunofluorescent findings on clinicopathological features in IgA nephropathy. Clin Exp Nephrol.

[13] Rizk D.V., Saha M.K., Hall S. (2019). Glomerular immunodeposits of patients with IgA nephropathy are enriched for IgG autoantibodies specific for galactose-deficient IgA1. J Am Soc Nephrol.

[14] Suzuki H., Fan R., Zhang Z. (2009). Aberrantly glycosylated IgA1 in IgA nephropathy patients is recognized by IgG antibodies with restricted heterogeneity. J Clin Invest.

[15] Moldoveanu Z., Suzuki H., Reily C. (2021). Experimental evidence of pathogenic role of IgG autoantibodies in IgA nephropathy. J Autoimmun.

[16] Novak L., Hall S.D., Cutter G. (2025). Kidney injury and colocalization of complement C3, IgA, and IgG in glomerular immune-complex deposits of patients with IgA nephropathy or IgA vasculitis with nephritis. Kidney Int.

[17] Barratt J., Lafayette R.A., Zhang H. (2023). IgA nephropathy: the lectin pathway and implications for targeted therapy. Kidney Int.

[18] Zhang H., Rizk D.V., Perkovic V. (2024). Results of a randomized double-blind placebo-controlled Phase 2 study propose iptacopan as an alternative complement pathway inhibitor for IgA nephropathy. Kidney Int.

[19] Lafayette R., Tumlin J., Fenoglio R. (2025). Efficacy and safety of Ravulizumab in IgA nephropathy: a Phase 2 randomized double-blind placebo-controlled trial. J Am Soc Nephrol.

[20] Perkovic V., Barratt J., Rovin B. (2025). Alternative complement pathway inhibition with iptacopan in IgA nephropathy. N Engl J Med.

[21] Mariani L.H., Bomback A.S., Canetta P.A. (2019). CureGN study rationale, design, and methods: establishing a large prospective observational study of glomerular disease. Am J Kidney Dis.

[22] Palmer M.B., Royal V., Jennette J.C. (2023). Cure glomerulonephropathy pathology classification and Core scoring criteria, reproducibility, and clinicopathologic correlations. Glomerular Dis.

[23] Trimarchi H., Barratt J., Cattran D.C. (2017). Oxford Classification of IgA nephropathy 2016: an update from the IgA Nephropathy Classification Working Group. Kidney Int.

[24] Pierce C.B., Munoz A., Ng D.K., Warady B.A., Furth S.L., Schwartz G.J. (2021). Age- and sex-dependent clinical equations to estimate glomerular filtration rates in children and young adults with chronic kidney disease. Kidney Int.

[25] Inker L.A., Eneanya N.D., Coresh J. (2021). New creatinine- and cystatin C-based equations to estimate GFR without race. N Engl J Med.

[26] Hara M., Endo Y., Nihei H., Hara S., Fukushima O., Mimura N. (1980). IgA nephropathy with subendothelial deposits. Virchows Arch A Pathol Anat Histol.

[27] Yang Y., Tang X., Yang Y. (2020). Glomerular C4 deposition and glomerulosclerosis predict worse renal outcomes in Chinese patients with IgA nephropathy. Ren Fail.

[28] Yang X., Yuan Y., Shao X. (2022). C4d as a screening Tool and an independent predictor of clinical outcomes in lupus nephritis and IgA nephropathy. Front Med (Lausanne).

[29] Wang Y., Jiang S., Di D. (2024). The prognostic role of activation of the complement pathways in the progression of advanced IgA nephropathy to end-stage renal disease. BMC Nephrol.

[30] Wu D., Li X., Yao X. (2021). Mesangial C3 deposition and serum C3 levels predict renal outcome in IgA nephropathy. Clin Exp Nephrol.

[31] Hiemstra P.S., Gorter A., Stuurman M.E., Van Es L.A., Daha M.R. (1987). Activation of the alternative pathway of complement by human serum IgA. Eur J Immunol.

[32] Hisano S., Matsushita M., Fujita T., Endo Y., Takebayashi S. (2001). Mesangial IgA2 deposits and lectin pathway-mediated complement activation in IgA glomerulonephritis. Am J Kidney Dis.

[33] Roos A., Rastaldi M.P., Calvaresi N. (2006). Glomerular activation of the lectin pathway of complement in IgA nephropathy is associated with more severe renal disease. J Am Soc Nephrol.

[34] Portilla D., Xavier S. (2021). Role of intracellular complement activation in kidney fibrosis. Br J Pharmacol.

[35] Zhou W., Marsh J.E., Sacks S.H. (2001). Intrarenal synthesis of complement. Kidney Int.

[36] Abe K., Miyazaki M., Koji T. (2001). Intraglomerular synthesis of complement C3 and its activation products in IgA nephropathy. Nephron.

[37] Segarra-Medrano A., Carnicer-Caceres C., Valtierra-Carmeno N. (2017). Study of the variables associated with local complement activation in IgA nephropathy. Nefrologia.

[38] Yu B.C., Park J.H., Lee K.H. (2022). Urinary C5b-9 as a prognostic marker in IgA nephropathy. J Clin Med.

[39] Wang Z., Xie X., Li J. (2021). Complement activation is associated with crescents in IgA nephropathy. Front Immunol.

[40] Dong Y., Wang Z., Guo W. (2024). Association between urinary C4d levels and disease progression in IgA nephropathy. Nephrol Dial Transplant.

[41] Nurmi M.S., Perez-Alos L., Garred P., Fellstrom B., Gabrysch K., Lundberg S. (2025). Urine complement-related proteins in IgA nephropathy and IgA vasculitis nephritis, possible biomarkers of disease activity. Clin Kidney J.

[42] Nangaku M., Pippin J., Couser W.G. (1999). Complement membrane attack complex (C5b-9) mediates interstitial disease in experimental nephrotic syndrome. J Am Soc Nephrol.

[43] Hsu S.I., Couser W.G. (2003). Chronic progression of tubulointerstitial damage in proteinuric renal disease is mediated by complement activation: a therapeutic role for complement inhibitors?. J Am Soc Nephrol.

[44] Wang S., Wu M., Chiriboga L. (2022). Membrane attack complex (MAC) deposition in renal tubules is associated with interstitial fibrosis and tubular atrophy: a pilot study. Lupus Sci Med.

[45] Bellur S.S., Lepeytre F., Vorobyeva O. (2017). Evidence from the Oxford Classification cohort supports the clinical value of subclassification of focal segmental glomerulosclerosis in IgA nephropathy. Kidney Int.

[46] Shankland S.J., Pippin J.W., Couser W.G. (1999). Complement (C5b-9) induces glomerular epithelial cell DNA synthesis but not proliferation in vitro. Kidney Int.

[47] Pippin J.W., Durvasula R., Petermann A., Hiromura K., Couser W.G., Shankland S.J. (2003). DNA damage is a novel response to sublytic complement C5b-9-induced injury in podocytes. J Clin Invest.

