# Supplementary material for: Clinical Value of Kidney Immunodeposits and Urinary Complement Activation Fragments in IgAN
Source: Kidney Int Rep. 2026 Jun 8;11(9):106648. doi: 10.1016/j.ekir.2026.106648 (PMC13382774; doi:10.1016/j.ekir.2026.106648)
Supplement: Supplemental file (PDF) — Figure S1. Associations between light microscopy and electron microscopy findings. Figure S2. Association between endocapillary hypercellularity and IgG intensity and localization by immunofluorescence. Figure S3. Univariate associations between pathology and survival from a combined event. Table S1. Associations between immunofluorescence and electron microscopy findings. Table S2. Associations between pathology findings by light microscopy and the subsequent use of immunosuppressive therapy. Table S3. Comparison of clinical features at measurement of tertiles of proteinuria and urinary sC5b9. STROBE checklist. [file mmc1.pdf]

Supplemental Material Table of Contents:

Table S1: Associations between immunofluorescence and electron microscopy findings

Table S2: Associations between pathology findings by light microscopy and the subsequent use of immunosuppressive therapy

Table S3. Comparison of clinical features at measurement of tertiles of proteinuria and urinary sC5b9.

Figure S1. Associations between light microscopy and electron microscopy findings

Figure S2. Association between endocapillary hypercellularity and IgG intensity and localization by immunofluorescence.

Figure S3: Univariate associations between pathology and survival from a combined event

STROBE checklist

Supplemental Table 1: Associations between immunofluorescence and electron microscopy findings

		IgA intensity IF	IgA localization IF	C3 intensity IF	C3 localization IF	IgG intensity IF	IgG localization IF	IgM intensity IF	IgM localization IF	Mesangial deposit EM	Subendo deposit EM
IgA intensity IF	Spearman’s ρ	-	0.27	0.589	0.391	0.252	0.214	0.18		0.347	0.148
	p		<.001	<.001	<.001	<.001	<.001	0.04		<.001	0.03
IgA localization IF	Spearman’s ρ		-	0.138	0.638	0.232	0.276	0.506		0.221	0.304
	p			0.03	<.001	<.001	<.001	<.001		<.001	<.001
C3 intensity IF	Spearman’s ρ			-	0.454	0.192	0.192			0.229	0.19
	p				<.001	0.003	0.003			<.001	0.004
C3 localization IF	Spearman’s ρ				-	0.321	0.394	0.44		0.231	0.254
	p					<.001	<.001	<.001		<.001	<.001
IgG intensity IF	Spearman ρ					-	0.929			0.221	
	p						<.001			<.001	
IgG localization IF	Spearman ρ						-	0.218		0.216	
	p							0.01		<.001	
IgM intensity IF	Spearman ρ							-			0.355
	p										<.001
IgM localization IF	Spearman ρ								-		
	p										
Mes deposit EM	Spearman ρ									-	0.418
	p										<.001

Legend: IF, immunofluorescence; EM, electron microscopy. Localization was codified as: 0, none; 1, mesangial only; 2, mesangial and capillary.

Supplemental Table 2: Associations between pathology findings by light microscopy and the subsequent use of immunosuppressive therapy

Pathology variable	Immunosuppression*	P value
Mesangial hypercellularity		
None	69	0.64
Focal (<50% of glomeruli)	65	
Diffuse (≥50% of glomeruli)	73	
Endocapillary hypercellularity (%)		
0	65	0.003
1-25	75	
26-50	92	
51+	93	
Segmental sclerosis (%)		
0	74	0.67
1-25	66	
26-50	74	
51+	63	
Tubular atrophy/ interstitial fibrosis (%)		
0	79	0.48
1-25	64	
26-50	70	
51+	71	
Crescents (%)		
0	66	<0.001
1-25	73	
26+**	100	

Legend: *Immunosuppression at any time from biopsy to end of follow-up. **Higher groups merged because of too few subjects. Trend tests were performed using Spearman's ρ.

Supplemental Table 3. Comparison of clinical features at measurement of tertiles of proteinuria and urinary sC5b9.

	Proteinuria				Urinary sC5b9			
	1st Tertile	2nd Tertile	3rd Tertile	P value	1st Tertile	2nd Tertile	3rd Tertile	P value
Female sex (%)	38	33	33	0.75	34	36	35	0.96
Age	34 ± 19	37 ± 18	36 ± 17	0.49	33 ± 17	37 ± 20	38 ± 17	0.14
eGFR	76 ± 30	71 ± 31	65 ± 33	0.07	74 ± 31	68 ± 30	68 ± 33	0.33
Prior IS (%)	54	56	68	0.12	60	58	59	0.95
Future IS (%)	26	51	74	<0.001	43	44	65	0.004

Legend: Proteinuria and urinary sC5b9 were creatinine ratios.

Supplemental Figure 1: Associations between light microscopy and electron microscopy findings

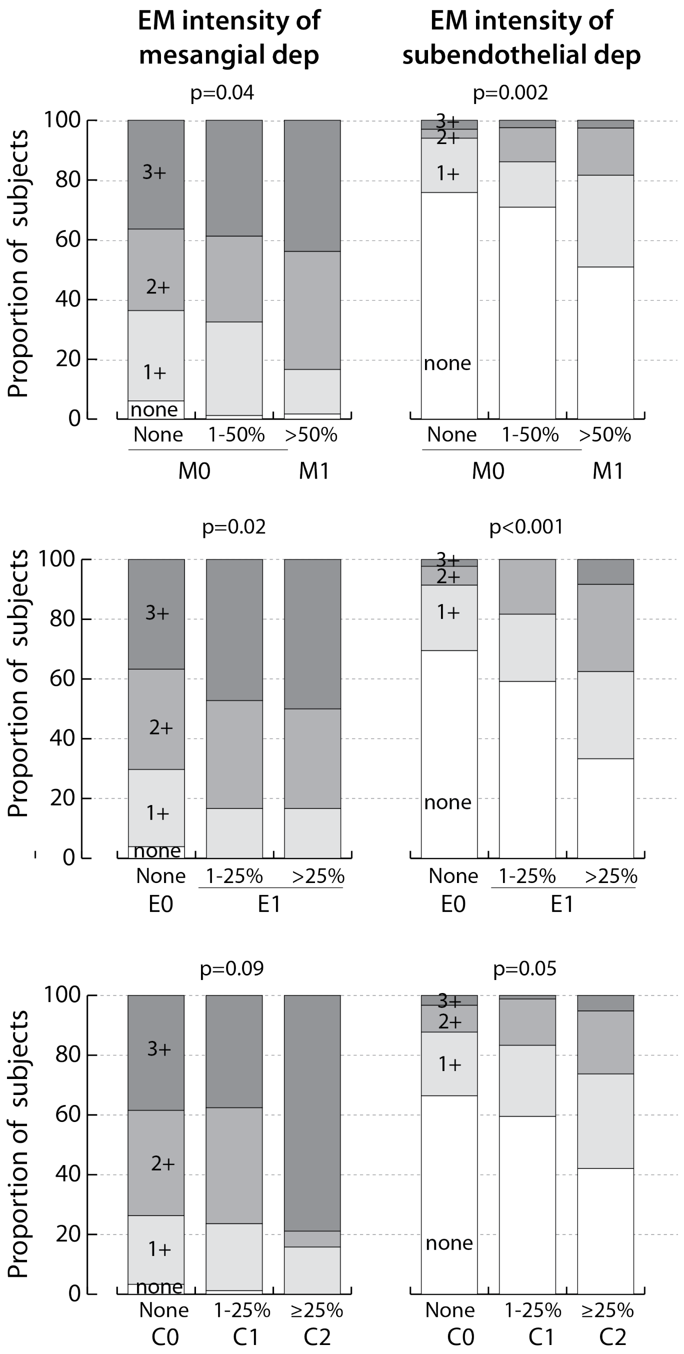

Supplemental Figure 2. Association between endocapillary hypercellularity and IgG intensity and localization by immunofluorescence.

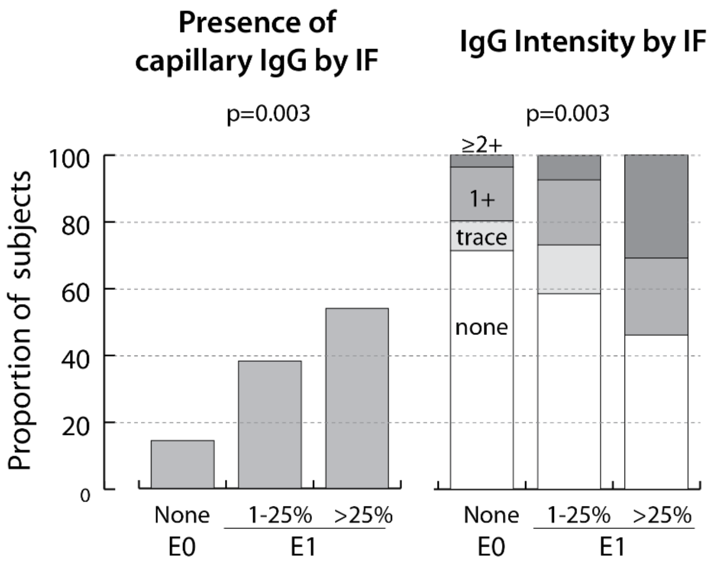

Legend: IF, immunofluorescence.

Supplemental Figure 3: Univariate associations between pathology and survival from a combined event

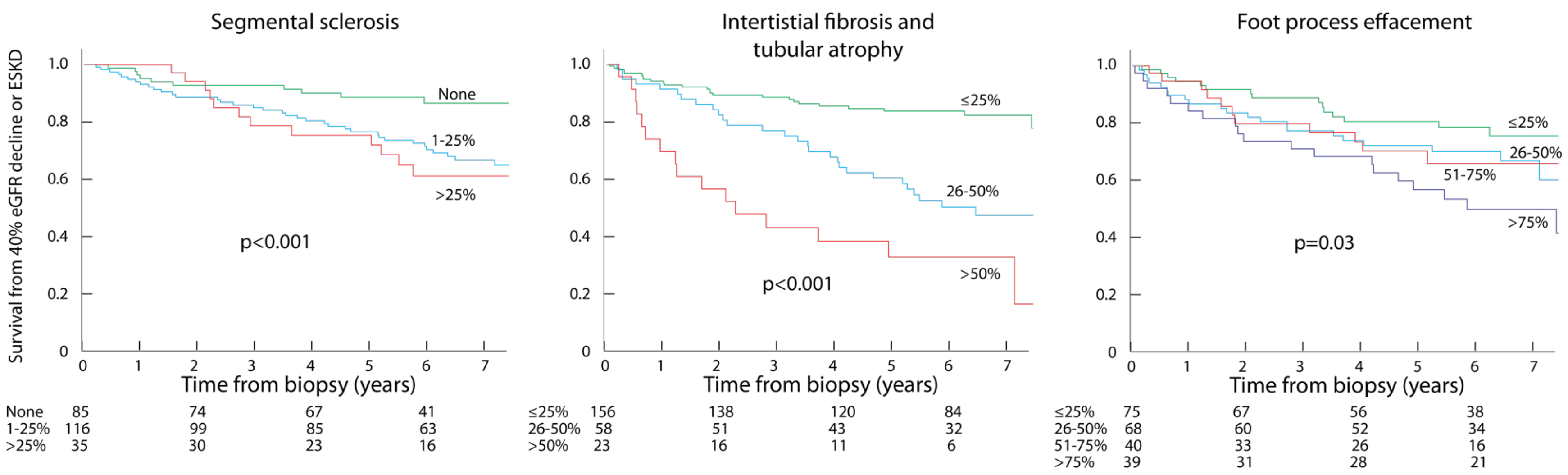

Legend: P values were trend tests obtained using Cox regression.

STROBE Statement—Checklist of items that should be included in reports of ***cohort studies***

	Item No	Recommendation	Page No
**Title and abstract**	1	(*a*) Indicate the study's design with a commonly used term in the title or the abstract<br>(*b*) Provide in the abstract an informative and balanced summary of what was done and what was found	Limited by #words<br>3
**Introduction**			
Background/rationale	2	Explain the scientific background and rationale for the investigation being reported	5
Objectives	3	State specific objectives, including any prespecified hypotheses	6
**Methods**			
Study design	4	Present key elements of study design early in the paper	7
Setting	5	Describe the setting, locations, and relevant dates, including periods of recruitment, exposure, follow-up, and data collection	7
Participants	6	(*a*) Give the eligibility criteria, and the sources and methods of selection of participants. Describe methods of follow-up<br>(*b*) For matched studies, give matching criteria and number of exposed and unexposed	7-9
Variables	7	Clearly define all outcomes, exposures, predictors, potential confounders, and effect modifiers. Give diagnostic criteria, if applicable	7-9
Data sources/ measurement	8*	For each variable of interest, give sources of data and details of methods of assessment (measurement). Describe comparability of assessment methods if there is more than one group	7-9
Bias	9	Describe any efforts to address potential sources of bias	7-9
Study size	10	Explain how the study size was arrived at	7
Quantitative variables	11	Explain how quantitative variables were handled in the analyses. If applicable, describe which groupings were chosen and why	7-9
Statistical methods	12	(*a*) Describe all statistical methods, including those used to control for confounding<br>(*b*) Describe any methods used to examine subgroups and interactions<br>(*c*) Explain how missing data were addressed<br>(*d*) If applicable, explain how loss to follow-up was addressed<br>(*e*) Describe any sensitivity analyses	10-11
**Results**			
Participants	13*	(a) Report numbers of individuals at each stage of study—eg numbers potentially eligible, examined for eligibility, confirmed eligible, included in the study, completing follow-up, and analysed<br>(b) Give reasons for non-participation at each stage<br>(c) Consider use of a flow diagram	12
Descriptive data	14*	(a) Give characteristics of study participants (eg demographic, clinical, social) and information on exposures and potential confounders<br>(b) Indicate number of participants with missing data for each variable of interest<br>(c) Summarise follow-up time (eg, average and total amount)	12-13
Outcome data	15*	Report numbers of outcome events or summary measures over time	12

Main results	16	(*a*) Give unadjusted estimates and, if applicable, confounder-adjusted estimates and their precision (eg, 95% confidence interval). Make clear which confounders were adjusted for and why they were included<br>(*b*) Report category boundaries when continuous variables were categorized<br>(*c*) If relevant, consider translating estimates of relative risk into absolute risk for a meaningful time period	12-15
Other analyses	17	Report other analyses done—eg analyses of subgroups and interactions, and sensitivity analyses	13-16
**Discussion**			
Key results	18	Summarise key results with reference to study objectives	17
Limitations	19	Discuss limitations of the study, taking into account sources of potential bias or imprecision. Discuss both direction and magnitude of any potential bias	17-19
Interpretation	20	Give a cautious overall interpretation of results considering objectives, limitations, multiplicity of analyses, results from similar studies, and other relevant evidence	17-19
Generalisability	21	Discuss the generalisability (external validity) of the study results	17-20
**Other information**			
Funding	22	Give the source of funding and the role of the funders for the present study and, if applicable, for the original study on which the present article is based	2

*Give information separately for exposed and unexposed groups.

Note: An Explanation and Elaboration article discusses each checklist item and gives methodological background and published examples of transparent reporting. The STROBE checklist is best used in conjunction with this article (freely available on the Web sites of PLoS Medicine at http://www.plosmedicine.org/, Annals of Internal Medicine at http://www.annals.org/, and Epidemiology at http://www.epidem.com/). Information on the STROBE Initiative is available at http://www.strobe-statement.org.
